# Delineating Diversity‐Based Freshwater Bioregions by Integrating Fish and Macroinvertebrates With Species Distribution Models and Spatial Clustering

**DOI:** 10.1002/ece3.72609

**Published:** 2025-12-08

**Authors:** Yajing He, Hongzhu Wang, Junyan Wu, Yongjing Zhao, Wenjuan Gao, Yongde Cui

**Affiliations:** ^1^ Institute of Hydrobiology Chinese Academy of Sciences Wuhan China

**Keywords:** aquatic biodiversity, beta diversity, bioregion delineation, conservation planning, freshwater ecosystem, MaxEnt

## Abstract

Delineating ecologically meaningful spatial units is fundamental for understanding biodiversity patterns and guiding effective conservation. Here, we delineated diversity‐based freshwater bioregions across the Yangtze River Basin (YRB) by integrating multi‐taxon biodiversity data with species distribution models and spatial clustering. We compiled distribution records of 391 fish species and 984 macroinvertebrate taxa across 13,716 hydrological units. MaxEnt models were used to predict species distributions based on key environmental drivers identified through principal component analysis and Mantel tests. Beta diversity was quantified using Jaccard dissimilarity, and non‐metric multidimensional scaling (NMDS) ordination was applied to extract the first three ordination axes for each taxon, which were then used in spatial clustering to define bioregions. Our approach identified four distinct bioregions (PERMANOVA, *R* ^2^ = 0.211, *p* < 0.001): Headwater and Western Sichuan Plateau (HWS), Hengduan Mountains–Sichuan Basin (HDS), Dabie–Qinling–Wuling Mountains (DQW), and Mid‐lower Floodplain (MLF). Fish and macroinvertebrates exhibited complementary distribution patterns, with fish preferring mainstem and primary tributaries, while macroinvertebrates favored streams and lakes. Beta diversity effectively revealed core transition zones including the southern edge of the Hengduan Mountains, the Sichuan Basin, the southeastern YRB margin, the estuary, and major tributary confluences. Compared to the scattered and discontinuous patterns generated by UPGMA clustering, spatial clustering preserved spatial connectivity and produced ecologically coherent bioregions. Some bioregions with lower species richness harbored relatively high beta diversity and proportions of threatened species, highlighting their conservation priority. This framework demonstrates how integrating multi‐taxon data with predictive modeling and spatial clustering can reduce data biases and provide an ecologically meaningful foundation for freshwater conservation planning, and it offers a practical tool for implementing bioregion‐specific management strategies.

## Graphical abstract

We integrated fish and macroinvertebrate data with species distribution models and spatial clustering to delineate coherent freshwater bioregions. The resulting bioregions captured complementary biodiversity patterns and key transition zones more effectively than single‐taxon or UPGMA clustering. This predictive multi‐taxon framework offers a robust tool for delineating spatially coherent bioregions and supporting freshwater conservation strategies.

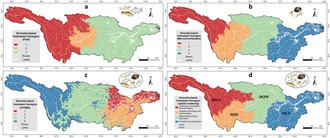

## 1 Introduction

Defining coherent and ecologically meaningful spatial units is essential for ecological research and conservation planning including priority setting, reserve design, and biodiversity monitoring (Lourie and Vincent 2004; Growns 2009; Baker and Hollowed 2014; Hill et al. 2020). By grouping areas with similar biological or ecological characteristics, such units can facilitate more effective conservation strategies and help resolve mismatches between protected area designation and actual biodiversity distributions (Woolley et al. 2020; Jupke et al. 2023; Haase et al. 2025). Numerous studies have delineated spatial units using terms such as ecoregions, ecological regions, biogeographic classifications, and bioregionalization (Abell et al. 2008; Baker and Hollowed 2014; Dinerstein et al. 2017; Hermogenes and Ebach 2020). These frameworks vary in focus, emphasizing species distribution patterns, environmental relationships, or historical and evolutionary processes. Here, we adopt the term “diversity‐based bioregion” to refer to regions that are relatively homogeneous in beta diversity, identified through spatial clustering with predictive distribution patterns.

Traditional bioregional delineation efforts have often relied on expert knowledge and focused on particular taxa (e.g., birds and mammals) or terrestrial ecosystem patterns (Caballero‐Herrera et al. 2022). For freshwater ecosystems, earlier broad‐scale frameworks made significant contributions by integrating the distribution of native freshwater fishes with watershed boundaries, geomorphological features, and expert knowledge (Abell et al. 2008). However, more detailed, region‐specific delineation is still needed to support fine‐scale conservation planning (Flitcroft et al. 2023). With advances in statistical methods, remote sensing, and machine learning, biogeographic delineation has increasingly shifted from qualitative to quantitative approaches (Ferrier et al. 2007; Kreft and Jetz 2010; Di Minin et al. 2022). Hill et al. (2020) reviewed various quantitative approaches, broadly classified into two‐stage approaches, including “Group First, then Predict” or “Predict First, then Group”, and one‐stage approaches. These approaches differ in methodology and their applicability across scales, taxonomic groups, and ecosystems. The “Group First, then Predict” method begins by clustering biological data into groups, followed by analyzing the relationship with environmental variables. The “Predict First, then Group” method uses environmental predictors first to construct species distribution models, followed by clustering to define bioregions. The “One‐Stage Approach” simultaneously analyzes biological groups and environmental relationships, particularly valuable for complex ecosystems with abundant data (Woolley et al. 2020). Two main grouping approaches are commonly used: non‐hierarchical algorithms, such as *k*‐means, and hierarchical methods, among which UPGMA (unweighted pair‐group method using arithmetic averages) has shown consistently strong performance (Kreft and Jetz 2010).

Despite advances in methodology, freshwater ecosystems continue to suffer disproportionately from severe biodiversity declines (Di Marco et al. 2017; Cowie et al. 2022). The Living Planet Index for freshwater populations has declined nearly four times more than that for terrestrial populations (WWF 2018; Reid et al. 2019). This decline is driven by intense anthropogenic pressures including habitat fragmentation, water pollution, overexploitation, and species invasion (Darwall et al. 2018; Haase et al. 2025). Moreover, freshwater conservation has received relatively less attention than terrestrial systems, with many protected areas failing to cover critical freshwater habitats (Jenkins et al. 2015; Jupke et al. 2023). Scientific research on freshwater species, especially invertebrates, remains limited, with many species listed as “data deficient” on the IUCN Red List (Dudgeon and Strayer 2024; IUCN 2024; Sayer et al. 2025). These specific threats and knowledge gaps may hinder the effective conservation of aquatic biodiversity.

Freshwater comprises less than 1% of the earth's surface yet supports almost 10% of all described species (Strayer and Dudgeon 2010; Sayer et al. 2025) including over half of all fish species (Carrete Vega and Wiens 2012). Fish and macroinvertebrates, which serve as excellent biodiversity surrogates, are crucial components of freshwater ecosystems (He et al. 2021). They occupy various trophic levels and functional niches, providing a comprehensive and complementary understanding of ecosystem structure and function. They also serve as essential food sources for higher trophic levels (Kang et al. 2014; Xing et al. 2016). Both groups have been extensively studied using standardized sampling methods and robust datasets, allowing for consistent and comparable analyses across regions (Feio et al. 2023). Bioregions defined by fish and macroinvertebrates better reflect ecological processes and species turnover than those based only on abiotic factors (Jupke et al. 2023; Sayer et al. 2025).

Currently, freshwater bioregional delineation mainly relies on species‐level information (Growns 2009; Leroy et al. 2019). However, beta diversity, which captures spatial compositional turnover among sites, provides additional insights beyond local species richness and has been increasingly applied for regionalization (Leprieur and Oikonomou 2014; Ye et al. 2019). Beta diversity is particularly valuable for identifying transition zones and biogeographic boundaries, as it directly reflects how communities change across environmental gradients and geographic distances (Hermogenes and Ebach 2020). In the Yangtze River Basin (YRB), existing bioregional research and conservation actions are mostly based on sub‐basins or administrative boundaries (Heiner et al. 2011; Huang et al. 2016). While operationally practical, these frameworks may not fully capture the complexity of biodiversity patterns. Moreover, previous bioregional studies in the YRB have focused predominantly on fish assemblages based on field survey data (He et al. 2020), which may introduce biases due to uneven spatial coverage, inconsistent taxonomic resolutions, and underrepresentation of rare or data‐deficient species (Brummitt and Lughadha 2003; Richardson and Whittaker 2010; Montalvo‐Mancheno et al. 2020).

Therefore, this study delineates diversity‐based freshwater bioregions by integrating multi‐taxon biodiversity with species distribution models and spatial clustering, thereby capturing comprehensive aquatic biodiversity patterns while preserving spatial connectivity. We first identified key environmental drivers for fish and macroinvertebrate groups using principal component analysis (PCA) and Mantel tests. MaxEnt models were then applied to predict potential species occurrence probabilities across hydrological units (HUs), which were subsequently converted into presence‐absence matrices. Beta diversity among HUs was quantified using Jaccard dissimilarity, and the pairwise matrices were ordinated through non‐metric multidimensional scaling (NMDS) to extract the first three axes of each taxon for clustering. To evaluate the effectiveness of multi‐taxon spatial clustering, we compared its results with those of fish‐only, macroinvertebrate‐only, and UPGMA hierarchical approaches. Finally, we examined community differences among bioregions using PERMANOVA and analyzed the functional and taxonomic compositions of fish and macroinvertebrates across bioregions. This predictive and spatial clustering framework provides an ecologically meaningful basis for freshwater biodiversity conservation by reducing data biases and integrating biodiversity patterns with spatial connectivity.

## 2 Materials and Methods

### 2.1 Hydrological Framework and River–Lake Network Model

The mainstem of the YRB is over 6300 km in length, with more than 10,000 tributaries and a total lake area of 20,714 km^2^. We divided the YRB into 13,716 hydrological units (HUs) with an average area of 123 km^2^ based on the HydroBASINS database (Lehner and Grill 2013). The hydrological framework served as the base layer for calculating the binary data and delineating the diversity‐based bioregions.

To construct a base map tailored for freshwater ecosystems used in species distribution modeling, we developed a river–lake network model that integrates HydroRIVERS (Lehner and Grill 2013) and HydroLAKES (Messager et al. 2016) databases. This approach ensures that aquatic habitats, rather than terrestrial grids, define the potential distributional space for freshwater species. We created hierarchical buffers for rivers based on stream order. According to the classification of river systems in China (Xu 2018), there are mainly five levels of rivers (mainstem, level 1; primary tributary, level 2; secondary tributary, level 3; tertiary tributary, level 4; quaternary tributary, level 5), with small rivers further divided into four levels (levels 6–9) according to Strahler's ordering system of HydroRIVERS. We measured the river width at various river levels using Google Earth measurements and validated them against records in the Encyclopedia of Rivers and Lakes in China (Editorial Committee of Encyclopedia of Rivers and Lakes in China 2010). Then, we established buffer distances of 0.5–1 km for the mainstem (level 1; 0.5 km for upper reaches, 1 km for mid–lower reaches), and 0.5, 0.25, 0.15, 0.1, 0.05, 0.025, 0.01, and 0.005 km for levels 2–9, respectively. We merged the river and lake polygon layers to form the river–lake network model, which serves as the base map for species distribution models (Appendix S1).

### 2.2 Distribution Data

For macroinvertebrates, a total of 637 sections (considering each lake and each river segment as a single section) were included in this study (Appendix S2), of which 284 sections of macroinvertebrate data were collected in the field by our research group (from 1980 to 2017) using D‐net, Surber net in the rivers, and Petersen grab in the lakes. To ensure the completeness and comprehensiveness of distribution data, 1030 rivers, 210 lakes, and 546 reservoirs in the YRB recorded in the book *Encyclopedia of Rivers and Lakes in China* (Editorial Committee of Encyclopedia of Rivers and Lakes in China 2010) were searched using macroinvertebrate‐related keywords such as “invertebrate”, “benthos”, “oligochaetes”, “mollusks”, and “aquatic insects”. A total of 298 articles from 1984 to 2017 were considered. Filtering peer‐reviewed studies with consistent methods, 143 articles were screened to acquire data. For macroinvertebrates, each taxonomic group (oligochaetes, leeches, polychaetes, mollusks, aquatic insects, crustaceans) was cross‐referenced with the latest monographs of Chinese taxonomic experts (prioritizing the Fauna Sinica) to ensure taxonomic harmonization. To ensure consistency and reduce potential bias caused by varying taxonomic levels from different sources, we standardized the data into genus (95%) and family (5%) levels for species distribution models. A small proportion of records could only be identified at the family level due to limited research on certain aquatic insect groups (e.g., Carabidae, Curculionidae, Blephariceridae). For many of these groups, taxonomic studies in China are still lacking, and most existing ecological studies only identify them at the family level.

The original data on fish distributions (235 sections) came from other authoritative teams of the Institute of Hydrobiology, Chinese Academy of Sciences, and partly (346 sections) from papers and books (1984–2017) (Appendix S2). Based on the Latin names of fish species, we verified synonyms on FishBase (https://fishbase.se/search.php) and had experts check our final fish checklist to ensure taxonomic harmonization. Fish functional traits included feeding habits (carnivore, omnivore, herbivore), migratory habits (migratory, native), and depth range (upper, lower, demersal). We compiled functional traits based on FishBase and Fish Traits Database (Frimpong and Angermeier 2009) or authoritative books (Chen 1998a; Chu et al. 1999; Yue 2000; S. Zhang 2001; Jing 2006; Wu and Zhong 2008; Zhang and Cao 2021). The threatened categories (CR, critically endangered; EN, endangered; VU, vulnerable; NT, near threatened; LC, least concern; DD, data deficient) are based on the book *China's Red List of Biodiversity: Vertebrates, Volume V, Freshwater Fishes (2021 edition)* (Zhang and Cao 2021).

Although our species distribution dataset spans an extended period (1984–2017), it is extremely challenging to obtain a comprehensive and up‐to‐date dataset covering the entire YRB. Importantly, over 80% of data were collected after 2010, particularly in human‐impacted regions such as the mainstem and large tributaries, where biodiversity monitoring efforts have been more intensive in recent years. In regions with sparse sampling or data gaps, mostly in remote or less‐disturbed tributaries, older records before 2010 were used to improve spatial coverage and reduce model bias.

### 2.3 Environmental Data

Environmental variables were classified into the following five groups, referencing previous studies (Esselman and Allan 2011; Heiner et al. 2011; Stein et al. 2014; Huang et al. 2016) (Appendix S3): (1) Bioclimatic variables including variables related to temperature and precipitation. Bio1–bio19 was obtained from the World Climate Database (http://www.worldclim.org) (Fick and Hijmans 2017). Water surplus and deficit were calculated as the difference between mean annual precipitation and mean annual evaporation, with evaporation data obtained from the DIVA‐GIS website (http://www.diva‐gis.org/). (2) Topographic variables, including elevation, slope, and aspect, were obtained from the Food and Agriculture Organization of the United Nations (http://www.fao.org). (3) Hydrological variables included hydrological unit (HU) area, waterbody type (He et al. 2021), downstream distance, upstream distance, and flow accumulation. Except for waterbody type, other original data were derived from HydroBASINS, HydroRIVERS, and HydroLAKES databases (Lehner and Grill 2013). (4) Land cover and population variables. This group was used to characterize the influence of human activities and disturbances on species distributions. Referring to a previous study on fish in the Jinsha River Basin (Sun et al. 2019), we established land cover variables separately for fish and macroinvertebrates. For fish, these included the proportion of forest, cultivated, grassland, shrubland, wetland, and waterbody cover. For macroinvertebrates, vegetation (forest, cultivated, grassland, shrubland) and water (wetland and waterbody) were aggregated into two categories. Additional variables included artificial cover, 20‐year variation of NDVI (1998–2018), and the population density per square kilometer. All data were obtained from the Resource and Environment Data Cloud Platform (http://www.resdc.cn), and land cover proportions were calculated for each hydrological unit. (5) Substrate variables, including topsoil texture and other characteristics, were obtained from the Harmonized World Soil Database (FAO/IIASA/ISRIC/ISSCAS/JRC 2012). The environmental rasters were clipped to the YRB layer (mask) at a 30‐arc‐second resolution and projected to the WGS‐1984 coordinate system.

The environmental variables of each section were extracted using the tool “extract by mask” in ArcGIS 10.6. Principal components analysis (PCA) was conducted separately within each variable group to reduce the dimensionality (Wei and Simko 2017). We retained principal components with a cumulative variance explanation exceeding 70% according to the scree plots. From each selected component, 1–3 variables with the highest loadings were extracted (see scree plots, biplots, and selected variable list in Appendices S4 and S5) (Huang et al. 2020). Mantel tests were then used to identify selected variables most correlated with species composition, ensuring that only ecologically meaningful predictors were included. Elevation and waterbody type were retained in all models due to their fundamental influence on aquatic biodiversity (Sun et al. 2019; He et al. 2021).

### 2.4 Species Distribution Models (SDMs)

Fish and macroinvertebrate distributions and corresponding high‐loading environmental variables were entered into MaxEnt (Elith and Leathwick 2009), a commonly used and reliable SDM for presence‐only data (Phillips et al. 2006). The model randomly selected 75% of the data for training and 25% for testing. In MaxEnt, the area under the curve (AUC) of the Receiver Operating Characteristic (ROC) curve was used to evaluate model accuracy. Models were acceptable when the AUCs were above 0.8. Then, we reclassified potential probability for each taxon from 0–0.5 to 0 and 0.5–1 to 1 in grid cells using the “reclassify” tool. Next, at the hydrological unit level, we calculated the total grid cell values for each macroinvertebrate genus and fish species using the “Zonal Statistics as Table” tool, where a higher value indicated greater suitability. Finally, the total values of hydrological units (HUs) were divided into five suitability grades using the quantile method in GIS. Grades 1–5 were divided by 20th, 40th, 60th, and 80th percentiles corresponded to extremely low (1), low (2), medium (3), high (4), and extremely high (5) suitability, respectively. Grades 4 and 5 were considered as potential presence (1), while Grades 1–3 were treated as absence (0), resulting in binary presence–absence for each HU. In addition, for species with < 10 sections or poor model performance, their actual distribution records were used as presence data directly. Further details on the models can be found in the ODMAP protocol of Appendix S11 (Zurell et al. 2020).

### 2.5 Beta Diversity

Beta diversity among hydrological units (HUs) was calculated using the Jaccard index with binary presence‐absence data derived from species distribution models. The Jaccard dissimilarity index was defined as *D*_*J*_ = (*b* + *c*)/(*a* + *b* + *c*), where *a* is the number of species present in both HUs, *b* is the number of species present only in the first HU, and *c* is the number of species present only in the second HU. The resulting dissimilarity values range from 0 (identical communities) to 1 (completely dissimilar communities). We calculated the index using the “vegdist” function (method = “jaccard”) in the vegan package, generating a full pairwise dissimilarity matrix for all HUs. This matrix was used for spatial clustering (Leprieur and Oikonomou 2014; Oikonomou et al. 2014; Ye et al. 2019). The mean pairwise Jaccard dissimilarity for each HU was calculated to map beta diversity patterns.

### 2.6 Delineating Diversity‐Based Freshwater Bioregions

Non‐metric multidimensional scaling (NMDS) was applied to Jaccard dissimilarities, and the first three axes of fish and macroinvertebrates were standardized and combined before being imported into GIS. Spatial clustering of the merged three‐dimensional NMDS coordinates for 13,716 hydrological units (HUs) was performed using the “Grouping Analysis” tool in ArcGIS to delineate freshwater bioregions. This method was selected because it incorporates beta diversity information while enforcing spatial contiguity, ensuring that the resulting bioregions are both ecologically coherent and spatially contiguous. The spatial clustering employed a *K*‐means algorithm with a spatial constraint based on *K*‐nearest neighbors (KNN, *K* = 8). The optimal number of clusters was identified using the pseudo *F*‐statistic (Appendix S7), which measures the ratio of between‐cluster variance to within‐cluster variance, with higher values indicating stronger clustering solutions. For comparison, spatial clustering was also applied separately to fish‐only and macroinvertebrate‐only matrices. In addition, we performed the unweighted pair group method with arithmetic mean (UPGMA), a frequently favored hierarchical clustering method, using the six‐dimensional NMDS axes of fish and macroinvertebrate assemblages (Kreft and Jetz 2010). The results of clusters were mapped to visualize their spatial distribution patterns. PERMANOVA (999 permutations) was used to test community metric differences among bioregions, based on standardized NMDS coordinates.

In addition, we compared the total species richness of fish and macroinvertebrates among different bioregions, as well as subgroup richness patterns. For fish, comparisons were made among feeding habits, migratory habits, and depth range. For macroinvertebrates, comparisons included taxonomic classes and functional feeding groups. All richness comparisons were conducted for significant differences among bioregions using pairwise Wilcoxon rank‐sum tests with adjusted *p*‐values, and visualized as raincloud plots combining half‐violin and boxplots to illustrate data distributions and median differences among bioregions. All statistical analyses were performed in R 4.3.3 (R Core Team 2024), and spatial visualizations were produced in ArcGIS 10.6 and ArcGIS Pro.

## 3 Results

### 3.1 Species Distribution Models

**FIGURE 1:**
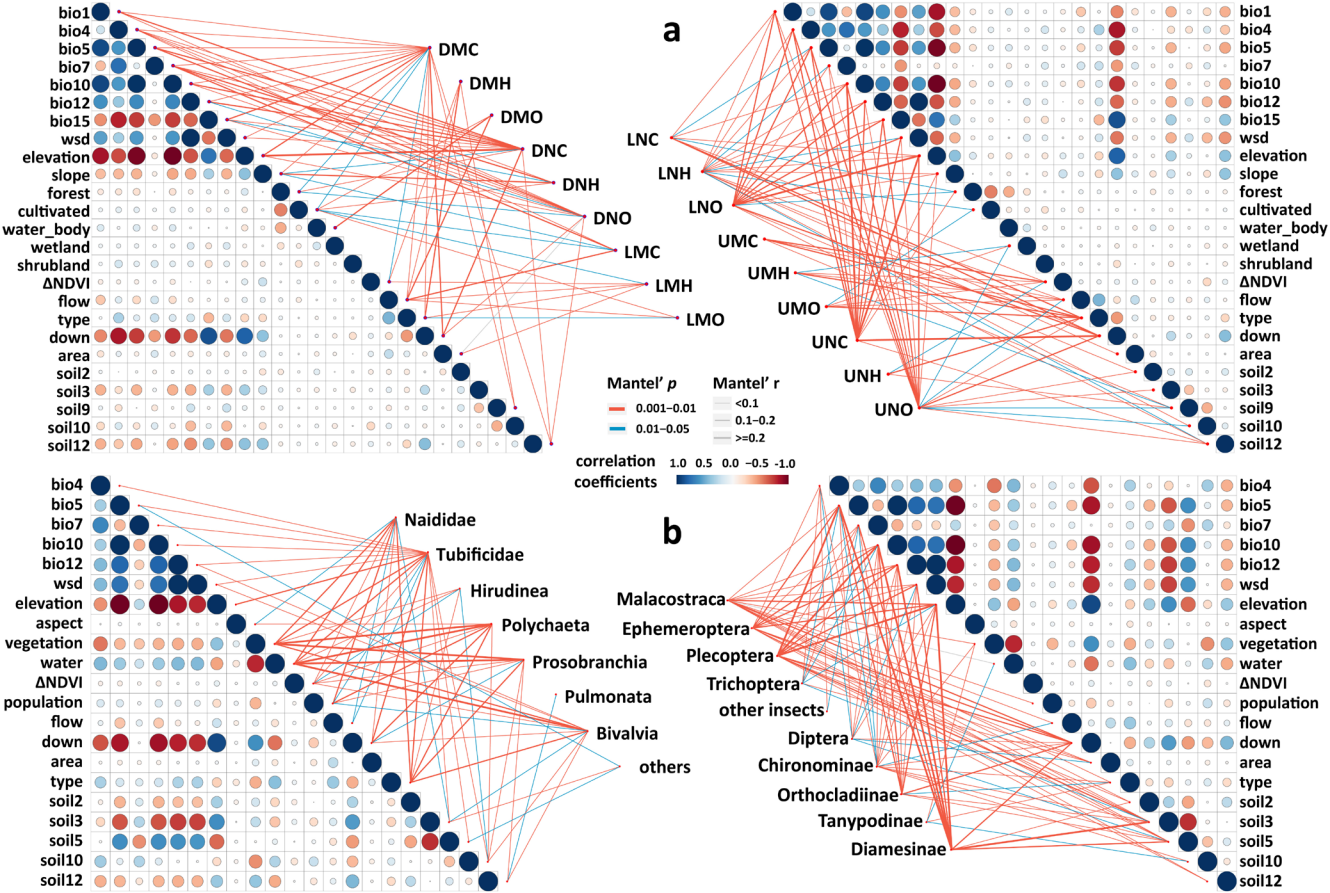
Pairwise comparisons of environmental variables with Spearman's correlation coefficients and relationships between community composition of macroinvertebrates (a) and fish (b) and environmental variables by the Mantel test. Line size and color gradient indicate Spearman's correlation coefficients. Line color shows statistical significance (*p*‐value), and line thickness represents Mantel's *r* statistic values. The full variable names are in Appendix S3. Fish functional groups, U: upper; L: lower; D: demersal; M: migratory; N: native; C: carnivore; O: omnivore; H: herbivore. bio1, Annual mean temperature; bio4, temperature seasonality; bio5, max temperature of warmest month; bio7, temperature annual range; bio10, mean temperature of warmest quarter; bio12, annual mean precipitation; bio15, precipitation seasonality (CV); wsd, water surplus and deficit; area, catchment area; type, water body type; down, distance from the reach outlet to the final downstream location; flow, flow accumulation; forest, cultivated, shrubland, wetland, water, representing the proportion of forest, cultivated, shrubland, wetland, water cover, respectively; population, population per km^2^; ΔNDVI, variation of NDVI during 20 years; soil2, topsoil gravel content; soil3, topsoil sand fraction; soil5, topsoil clay fraction; soil9, topsoil CEC (clay); soil10, topsoil CEC (soil); soil12, topsoil calcium carbonate. See details in Section 2.3 and Appendix S3.

A total of 391 fish species in 141 genera, 35 families, and 15 orders were recorded in the YRB. A total of 984 taxa of macroinvertebrates belonging to 608 genera, 181 families, 32 orders, 9 classes, and 5 phyla were included. According to the results of the principal component analysis, 21 and 25 variables were selected for macroinvertebrates and fish, respectively. The Mantel test (*p* < 0.05) revealed the key factors for each group of macroinvertebrates and fish (Figure 1). In general, land cover variables strongly influenced annelids and mollusks, while bioclimatic and topographic variables were more important for arthropods (Figure 1a). Bioclimatic, topographic, and hydrological variables were more important to fish (Figure 1b). Selected variables for each group are shown in Appendix S6.

366 macroinvertebrate genera met the modeling requirements, among which 357 genera showed good modeling performance, while 9 genera failed (training AUC = 0.934 ± 0.003; test AUC = 0.837 ± 0.031). Among the 391 fish species, 296 met the modeling requirements, and models were successfully built for 288 species. The average training and test AUCs were 0.970 ± 0.029 and 0.911 ± 0.056, respectively. For species with fewer than 10 sections and for which models failed, their distributions across hydrological units were directly assigned based on the observed presence of sections.

### 3.2 Fish and Macroinvertebrate Spatial Patterns

**FIGURE 2:**
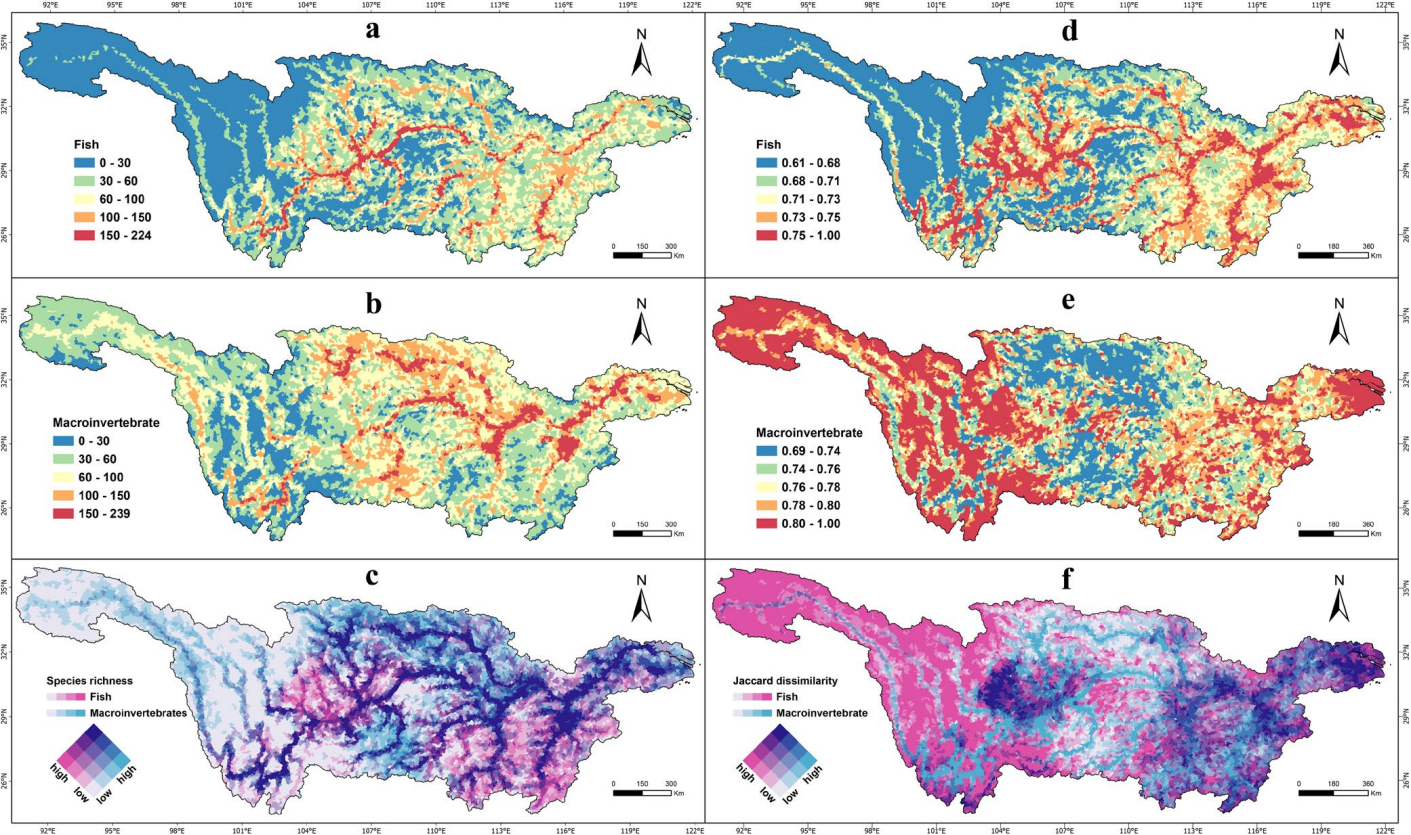
Spatial patterns of species richness (left panels) and Jaccard dissimilarity (right panels) for fish and macroinvertebrates across the Yangtze River Basin. (a, b) Species richness of fish and macroinvertebrates; (c) bivariate map of combined species richness; (d, e) Jaccard dissimilarity of fish and macroinvertebrates; (f) bivariate map of combined Jaccard dissimilarity.

Based on the spatial patterns of species richness (Figure 2a–c), areas with high fish diversity were mainly distributed along the mainstem, especially from the Lijiang to Yichang reaches, and in primary tributaries. High richness of macroinvertebrates occurred primarily in the middle and lower reaches, particularly within the potamo‐lacustrine complex ecosystem, as well as in structurally diverse tributaries, small streams, and confluence zones. The mainstem below Lijiang, together with the mid‐lower reaches of the Jialing River Basin, Wu River Basin, and the mid‐lower floodplain, represented biodiversity hotspots for both fish and macroinvertebrates.

Beta diversity patterns (Figure 2d–f) showed high values for fish in the mainstem and key tributaries of the Yunnan–Guizhou Plateau, the Sichuan Basin, and the Poyang and Dongting Lake Basins. Macroinvertebrate beta diversity was highest in the headwaters, the Jinsha River Basin, and several scattered streams and lakes of the mid‐lower YRB. Although the two groups showed different spatial patterns, regions with high beta diversity for both were located in parts of the tributaries within the Yunnan–Guizhou Plateau, the Sichuan Basin, and the large lake basins such as Dongting, Poyang, and Taihu. These regions are characterized by complex topography and dense river networks.

### 3.3 Diversity‐Based Freshwater Bioregion Delineation

To evaluate the advantages of integrated multi‐taxon spatial clustering for bioregional delineation, we compared the results from fish‐only spatial clustering, macroinvertebrate‐only spatial clustering, and the traditional UPGMA method.

**FIGURE 3:**
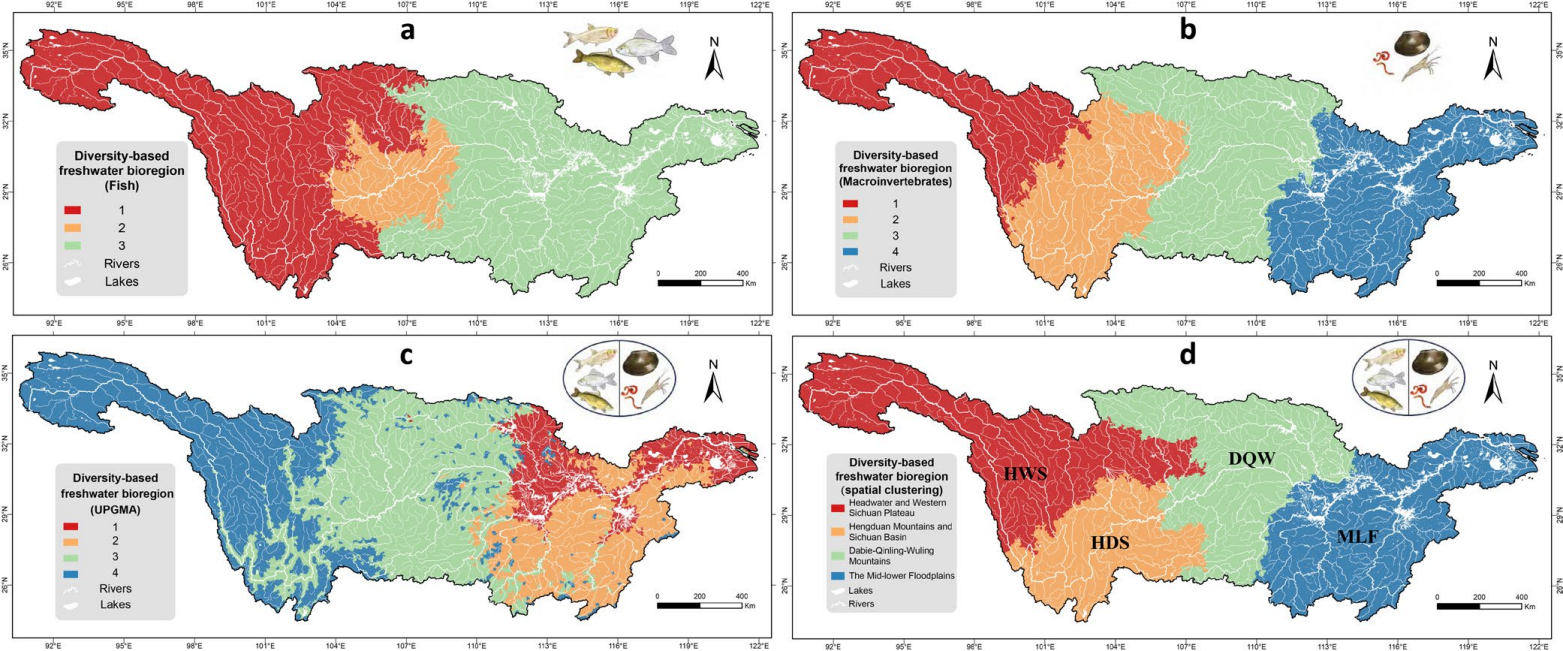
Comparison of bioregion delineation schemes for the Yangtze River Basin. (a) Fish‐only spatial clustering (3 bioregions), (b) macroinvertebrate‐only spatial clustering (4 bioregions), (c) traditional UPGMA clustering, and (d) integrated multi‐taxon spatial clustering (4 bioregions: DQW, Dabie–Qinling–Wuling Mountains; HDS, Hengduan Mountains‐Sichuan Basin; HWS, Headwater and Western Sichuan Plateau; MLF, Mid‐lower Floodplain region).

Fish‐only spatial clustering identified three bioregions (pseudo‐*F* = 2266.84, Appendix S7a and Figure 3a), exhibiting a distinct east–west (1–3) differentiation pattern with the Sichuan Basin (2) forming a transitional zone. Macroinvertebrate‐only spatial clustering identified four optimal bioregions (pseudo‐*F* = 2644.99; Appendix S7b and Figure 3b), sequentially from upstream to downstream: headwater and upper Yalong River region (1), Yunnan‐Guizhou Plateau and Sichuan Basin transition zone (2), central mainstem connection zone (3), and mid‐lower floodplain core area (4). However, the UPGMA method generated four regions and exhibited significant spatial fragmentation, forming numerous scattered patches and isolated fragments inconsistent with the hydrological connectivity of freshwater ecosystems (Figure 3c). Details of the UPGMA clustering and NMDS ordination of fish and macroinvertebrates are shown in Appendix S8.

The integrated multi‐taxon spatial clustering identified four optimal bioregions (pseudo‐*F* = 1871.38; Appendix S7c and Figure 3d), delineated as four diversity‐based freshwater bioregions: Headwater and Western Sichuan Plateau (HWS), Hengduan Mountains–Sichuan Basin (HDS), Dabie–Qinling–Wuling Mountains (DQW), and Mid‐lower Floodplain (MLF). This scheme provides an organic integration of spatial continuity and hydrological connectivity while preserving multi‐taxon biodiversity patterns.

### 3.4 Differences Between Bioregions

Community composition differed significantly among the four bioregions (PERMANOVA, pseudo‐*F* = 3673.20, *R* ^2^ = 0.211, *p* = 0.001; Appendix S9). Pairwise PERMANOVA tests further revealed significant differences between all pairs of bioregions (all adjusted *p* < 0.001), with the greatest compositional dissimilarity observed between HWS and MLF (*R* ^2^ = 0.335), and the smallest between HDS and DQW (*R* ^2^ = 0.074).

**FIGURE 4:**
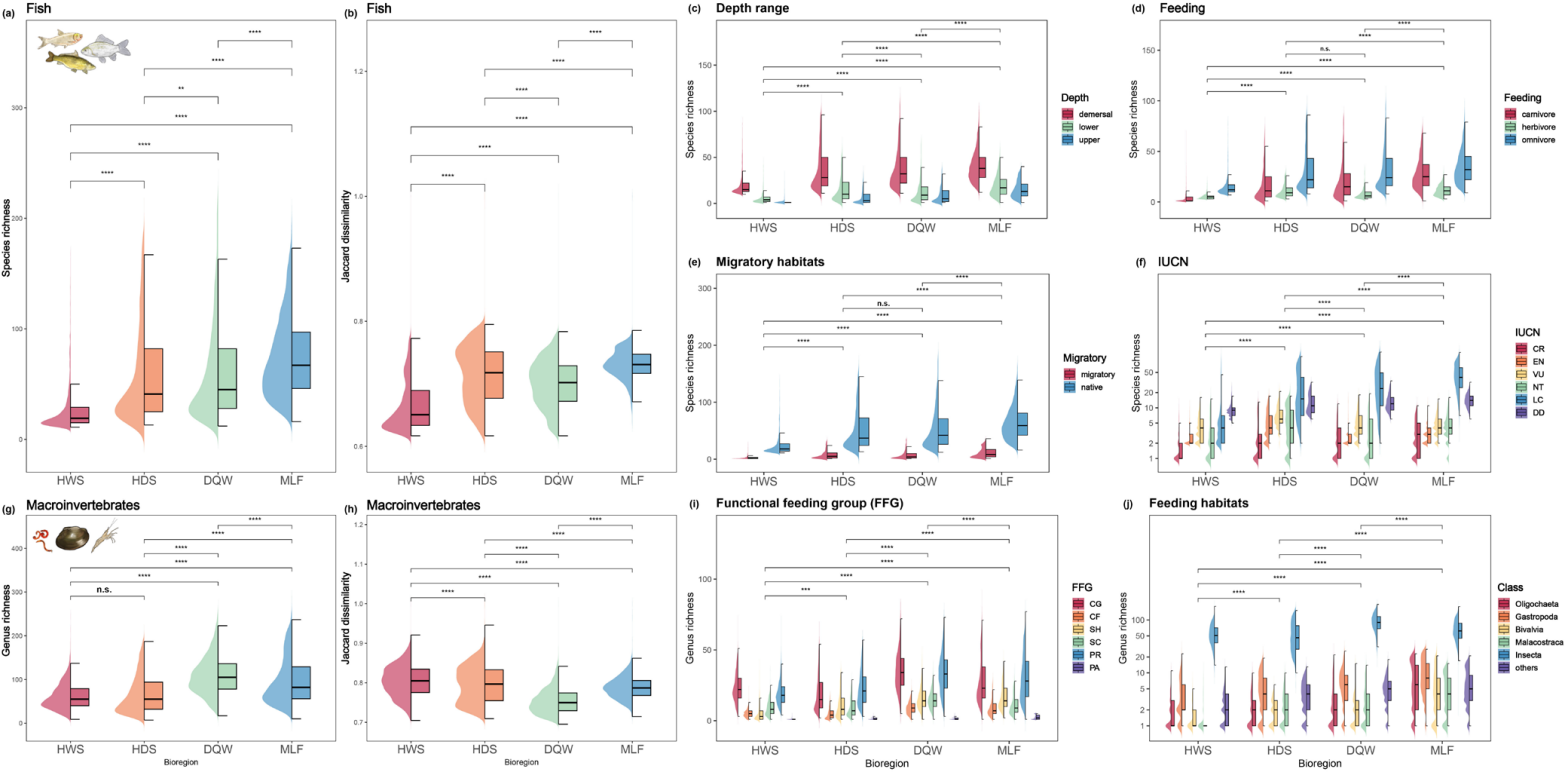
Total richness, Jaccard dissimilarity, and composition of different taxonomic and functional groups for fish species and macroinvertebrate genera across four bioregions. (a–f) Fish species: (a) total richness, (b) Jaccard dissimilarity, (c) depth range, (d) feeding habits, (e) migratory habits, and (f) IUCN status. (g–j) Macroinvertebrate genera: (g) total richness, (h) Jaccard dissimilarity, (i) functional feeding groups, and (j) taxonomic classes. CR, Critically Endangered; EN, Endangered; VU, Vulnerable; NT, Near Threatened; LC, Least Concern; DD, Data Deficient. CG, collector‐gatherer; CF, collector‐filterer; SH, shredder; SC, scraper; PR, predator; PA, parasite.

Fish species richness showed an increasing trend from upstream to downstream bioregions (Figure 4a). The mid‐lower floodplain (MLF) bioregion exhibited the highest biodiversity, characterized by the high total fish richness and Jaccard dissimilarity, and the most abundant carnivorous and migratory assemblages (Figure 4a–e). In contrast, the HWS bioregion had the lowest species richness and relatively simple ecological functional compositions, whereas its macroinvertebrate assemblages displayed the highest Jaccard dissimilarity, suggesting a strong spatial turnover despite low local richness. The Hengduan Mountains and Sichuan Basin (HDS) and Dabie–Qinling–Wuling Mountains (DQW) bioregions showed intermediate richness but particularly high Jaccard dissimilarity in both fish and macroinvertebrates, as well as higher diversity of demersal, omnivorous, and native fish species (Figure 4c–e,g). Notably, HDS contained the highest number of IUCN threatened species (Figure 4f) and exhibited relatively high Jaccard dissimilarity in both groups, highlighting its critical importance for biodiversity conservation. Across all bioregions, macroinvertebrates were dominated by gatherers‐collectors (GC) and predators (PR), with Insecta being overwhelmingly dominant, while the proportions of Oligochaeta, Gastropoda, Bivalvia, and Malacostraca increased significantly in the MLF (Figure 4i,j). Except for non‐significant differences in fish feeding habits between HDS and DQW and macroinvertebrate genus richness between HWS and HDS, other pairwise comparisons among bioregions showed highly significant differences (*p* < 0.001).

## 4 Discussion

### 4.1 Complementary Patterns and the Value of Beta Diversity

Fish and macroinvertebrates are widely regarded as the most effective surrogates for indicating freshwater ecosystem health (Feio et al. 2023) and exhibit complementary distribution patterns driven by different environmental factors. Macroinvertebrates are mainly influenced by local landscape and environmental factors such as land cover and water quality (Strayer and Dudgeon 2010; Collier et al. 2016) (Figure 1b), while fish are driven predominantly by broader‐scale factors, including climate, topography, and hydrological connectivity (Reid et al. 2019) (Figure 1a). Consequently, fish tend to occupy mainstem rivers and large tributaries, whereas macroinvertebrates are more abundant in streams and lakes (Figure 2a,b,d,e). These complementary patterns reflect differences in ecological traits and life‐history strategies, emphasizing the importance of multi‐taxon approaches in biodiversity assessment and conservation planning.

Recent advancements in large‐scale distribution databases, geospatial techniques, and computational power have facilitated the quantitative delineation of freshwater bioregions globally (Dudgeon et al. 2006; Meyer et al. 2015). Several studies focusing on fish at the global, Chinese, or basin scales have identified biogeographic boundaries in the YRB, typically located between its upper and mid‐lower reaches (Zhang 1954; Olson and Dinerstein 2002; Kang et al. 2014), or near the upper Jinsha River and the mid‐lower Yalong River (Li 1981; Abell et al. 2008; He et al. 2020), or divided by the Hengduan Mountains (Chen 1998b; Kang et al. 2014; Sun et al. 2019), reflecting the historical barrier effects of these regions. Consistent with these results, our study reveals that the HWS and the Hengduan Mountains and Sichuan Basin (HDS) bioregions are particularly distinctive (Figures 2 and 3d). Further analysis indicates distinct high Jaccard dissimilarity, functional assemblages, and high proportions of endangered fish species in HDS (Figure 4b,f,h,j), demonstrating that regions with fewer species can still be critically significant for biodiversity conservation through compositional uniqueness and potential endemism (Jansen et al. 2022).

Beta diversity, which captures spatial compositional turnover among sites, provides additional insights beyond local species richness (Leprieur and Oikonomou 2014). In our study, beta diversity effectively revealed several transition zones, including the southern edge of the Hengduan Mountains, the Sichuan Basin, the southeastern margin of the YRB, and the estuary, as well as confluence zones where major tributaries join the Yangtze mainstem (Figure 2d–f). Transition zones typically host high compositional turnover, leading to biological spillover effects and often serve as biodiversity hotspots (Morrone 2015; Leroy et al. 2019; Hermogenes and Ebach 2020; Dias et al. 2024).

These complementary patterns and differences between these two taxonomic groups highlight the value of beta diversity in recognizing underlying ecological processes better. However, several issues warrant further consideration. First, boundary delineation exhibits scale dependency (Brown et al. 2015; Sayer et al. 2025). Second, anthropogenic barriers such as dams can alter these patterns by fragmenting river networks and impeding dispersal (Barbarossa et al. 2020). Incorporating spatial data on dam locations and hydrological alterations in future research could help refine our understanding of human impacts on freshwater biodiversity patterns and boundaries.

### 4.2 Suitable Bioregional Delineation for Freshwater Ecosystem and Biodiversity Conservation

Effective conservation requires spatially explicit bioregions that align with actual biodiversity distributions and support objective‐driven management decisions (Brown et al. 2015; Jenkins et al. 2015). The delineation process mainly depends on the targeted taxa, the evenness of data coverage, and the classification algorithms applied (Woolley et al. 2020; Sayer et al. 2025). The “two‐stage” framework offers two main strategies: “Group First, then Predict” and “Predict First, then Group” (Hill et al. 2020). The former is more suitable for studies focusing on well‐defined biological groups, but may overlook complex biological‐environmental interactions (Zhang et al. 2019; He et al. 2020; Pamungkas et al. 2021). In contrast, the latter approach effectively incorporates environmental gradients (Stephenson et al. 2018; Tittensor et al. 2019) and is increasingly recognized for capturing large‐scale biodiversity trends and spatial patterns (Leroy et al. 2023), particularly with presence‐only data (Fourcade et al. 2018).

Our study aimed to analyze biodiversity patterns on a whole‐basin scale using presence‐only multi‐taxon data, making the “Predict First, then Group” method more appropriate. Previous studies on the freshwater bioregion/ecoregion delineation in the YRB primarily relied on field investigation data and focused solely on fish species (Abell et al. 2008; Xing et al. 2016; He et al. 2020), potentially introducing biases from uneven data coverage. By applying statistical processes and species distribution models tailored to the freshwater ecosystem, our study transformed the high suitability in hydrological units into presence‐absence data, thereby compensating for incomplete survey coverage. To improve model accuracy, we applied different model parameters for macroinvertebrate and fish groups (Figure 1; Appendix S6). Additionally, we included the distribution points of rare species and those with fewer distribution sites directly into the spatial clustering analysis to avoid overfitting.

Many studies have traditionally employed hierarchical clustering methods such as the unweighted pair group method with arithmetic mean (UPGMA) to delineate bioregions based on species composition or environmental similarity (Kreft and Jetz 2010; Ye et al. 2019; He et al. 2020). Compared with the fragmented patterns generated by UPGMA clustering (Figure 3c), the spatial clustering (Figure 3d) method ensures that neighboring hydrological units are grouped into spatially continuous bioregions (Miele et al. 2014). This approach effectively preserves the connectivity of hydrological units in freshwater ecosystems, an essential condition for species dispersal, migration, and community assembly (Tonkin et al. 2018). By integrating biological dissimilarity with ecological integrity, the spatial clustering produces bioregions that better correspond to natural watershed boundaries and ecological processes.

Conservation and management in the YRB have often been implemented at the level of administrative provinces or sub‐basins (Kang et al. 2014; Huang et al. 2016) (Appendix S10). However, these ecoregions manage the upper, middle, and lower reaches as an integrated ecosystem, overlooking their distinct conservation needs. According to the river continuum concept, the physical and biological conditions change gradually from headwaters to the river mouth, forming a continuous ecological gradient (Vannote et al. 1980; Doretto et al. 2020). Our delineation demonstrated that several sub‐basins were further divided into distinct diversity‐based bioregions (Figure 2 and Appendix S10). For example, the Jinshajiang and Mintuo Sub‐basins were divided into upper reaches belonging to the HWS (Headwater and Western Sichuan Plateau bioregion) and lower reaches classified as HDS (Hengduan Mountains and Sichuan Basin bioregion). Similarly, the Wujiang and Upper Mainstem sub‐basins were divided between HDS in the upstream and DQW (Dabie–Qinling–Wuling Mountains bioregion) in the downstream, while the western part of the Lake Dongting Basin was assigned to DQW and the eastern part to MLF (The Mid‐lower Floodplains bioregion). These divisions better capture the combined influences of topography, climate, and hydrological connectivity on biodiversity distribution, and are consistent with the river continuum concept, highlighting the ecological rationale of our clusters.

Defining bioregions and planning conservation actions based on biodiversity patterns can greatly improve the efficiency and representativeness of the protected area network (Adams et al. 2019). The HWS bioregion, with low species richness but unique high‐altitude fauna, requires strict protection of headwater habitats and climate change monitoring. The HDS bioregion, harboring the highest proportion of threatened species and high beta diversity, needs urgent establishment of protected areas and habitat restoration to safeguard native and endemic species. The DQW bioregion, supporting diverse functional assemblages, should balance biodiversity conservation with sustainable resource use through seasonal fishing closures and riparian restoration. The MLF bioregion, exhibiting the highest species richness and supporting critical migratory populations, requires floodplain connectivity restoration and integrated watershed management to address intensive anthropogenic pressures. Additionally, transition zones with high beta diversity for both taxa should be prioritized as ecological corridors to maintain longitudinal connectivity.

Our framework offers several advantages over traditional bioregionalization methods. First, by integrating fish and macroinvertebrate assemblages with species distribution models (SDMs) to reduce data gaps and uneven sampling, it captures complementary biodiversity patterns that single‐taxon approaches often overlook (Figure 2). Second, using the river–lake network as the SDM base map and hydrological units as clustering units, the spatially constrained clustering preserves hydrological connectivity and ecological coherence, improving upon the fragmented patterns generated by UPGMA (Figure 3c vs. 3d). Third, applying beta diversity for delineation not only identifies ecological transition zones but also captures species turnover among regions, revealing underlying community assembly processes beyond simple richness patterns (Figures 2f, 3d, 4).

While this study demonstrates the value of predictive modeling for freshwater bioregion delineation, several limitations should be acknowledged. The combination of modeled and observed data may introduce bias, particularly for rare or data‐deficient species. Even with well‐calibrated MaxEnt models, presence‐only data may still produce biased predictions, and spatial autocorrelation between training and testing datasets may inflate model evaluation metrics such as AUC. Future studies could consider alternative strategies, including rarity‐weighted indices, modeling methods specifically optimized for sparse data, or spatially explicit cross‐validation techniques to address spatial autocorrelation better. Nonetheless, this study provides a practical framework integrating multi‐taxon biodiversity data with species distribution models and spatial clustering for freshwater bioregion delineation, contributing to more ecologically representative conservation planning.

## Author Contributions

**Yajing He:** conceptualization (lead), formal analysis (lead), investigation (equal), methodology (lead), visualization (lead), writing – original draft (lead), writing – review and editing (lead). **Hongzhu Wang:** conceptualization (lead), funding acquisition (equal), supervision (equal), writing – review and editing (equal). **Junyan Wu:** formal analysis (equal), investigation (equal), visualization (equal), writing – review and editing (equal). **Yongjing Zhao:** data curation (equal), investigation (equal), project administration (equal), writing – review and editing (equal). **Wenjuan Gao:** data curation (equal), investigation (equal), project administration (equal), writing – review and editing (equal). **Yongde Cui:** conceptualization (lead), funding acquisition (lead), supervision (lead), writing – review and editing (equal).

## Funding

This study was supported by the National Key Research and Development Program of China (2021YFC3200103), the National Natural Science Foundation of China (52039006) and the Project of Yellow River Fisheries Resources and Environment Investigation from the MARA, P.R. China (HHDC‐2022‐05).

## Conflicts of Interest

The authors declare no conflicts of interest.

## Supporting information


**Appendix S1:** Hydrological network of rivers and lakes in the Yangtze River Basin.
**Appendix S2:** Study sections of fish (black dots) and macrozoobenthos (orange dots) in the Yangtze River Basin.
**Appendix S3:** Environmental variables prepared for species distribution models (table).
**Appendix S4:** Scree plots and biplots in the principal component analysis of five environmental groups for fish.
**Appendix S5:** Scree plots and biplots in principal component analysis of five environmental groups for macroinvertebrates.
**Appendix S6:** Selecting environmental variables for each group in the MaxEnt model.
**Appendix S7:** The relationship between cluster numbers 2 through 15 and pseudo *F*‐statistic.
**Appendix S8:** UPGMA clustering and NMDS ordination.
**Appendix S9:** Overall PERMANOVA and pairwise PERMANOVA tests based on Bray–Curtis dissimilarities among the four groups.
**Appendix S10:** Comparison of provincial boundaries, sub‐basin boundaries, and the bioregional delineation in this study.
**Appendix S11:** The ODMAP protocol of this study.

## Data Availability

The authors confirm that some data supporting the findings of this study are available within the article and the Supporting Information. Additional materials may be obtained from the corresponding author upon reasonable request.
