# Supplementary material for: Delineating Diversity‐Based Freshwater Bioregions by Integrating Fish and Macroinvertebrates With Species Distribution Models and Spatial Clustering
Source: Ecol Evol. 2025 Dec 8;15(12):e72609. doi: 10.1002/ece3.72609 (PMC12683362; doi:10.1002/ece3.72609)
Supplement: Supplementary file 1 — Appendix S1: Hydrological network of rivers and lakes in the Yangtze River Basin. Appendix S2: Study sections of fish (black dots) and macrozoobenthos (orange dots) in the Yangtze River Basin. Appendix S3: Environmental variables prepared for species distribution models (table). Appendix S4: Scree plots and biplots in the principal component analysis of five environmental groups for fish. Appendix S5: Scree plots and biplots in principal component analysis of five environmental groups for macroinvertebrates. Appendix S6: Selecting environmental variables for each group in the MaxEnt model. Appendix S7: The relationship between cluster numbers 2 through 15 and pseudo F‐statistic. Appendix S8: UPGMA clustering and NMDS ordination. Appendix S9: Overall PERMANOVA and pairwise PERMANOVA tests based on Bray–Curtis dissimilarities among the four groups. Appendix S10: Comparison of provincial boundaries, sub‐basin boundaries, and the bioregional delineation in this study. Appendix S11: The ODMAP protocol of this study. [file ECE3-15-e72609-s001.zip › Appendices 1-10.docx]

### Appendix 1


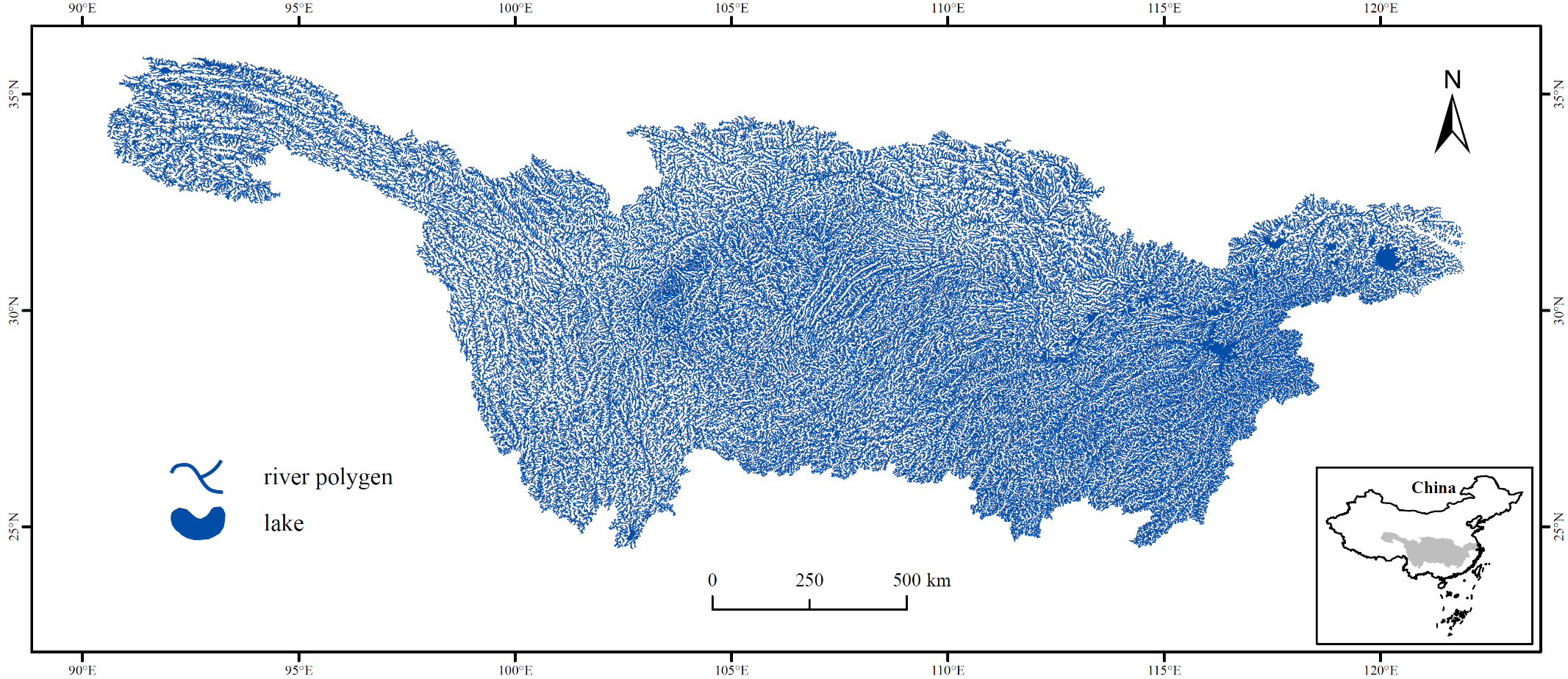


**Appendix 1** Hydrological network of rivers and lakes in the Yangtze River Basin

### Appendix 2


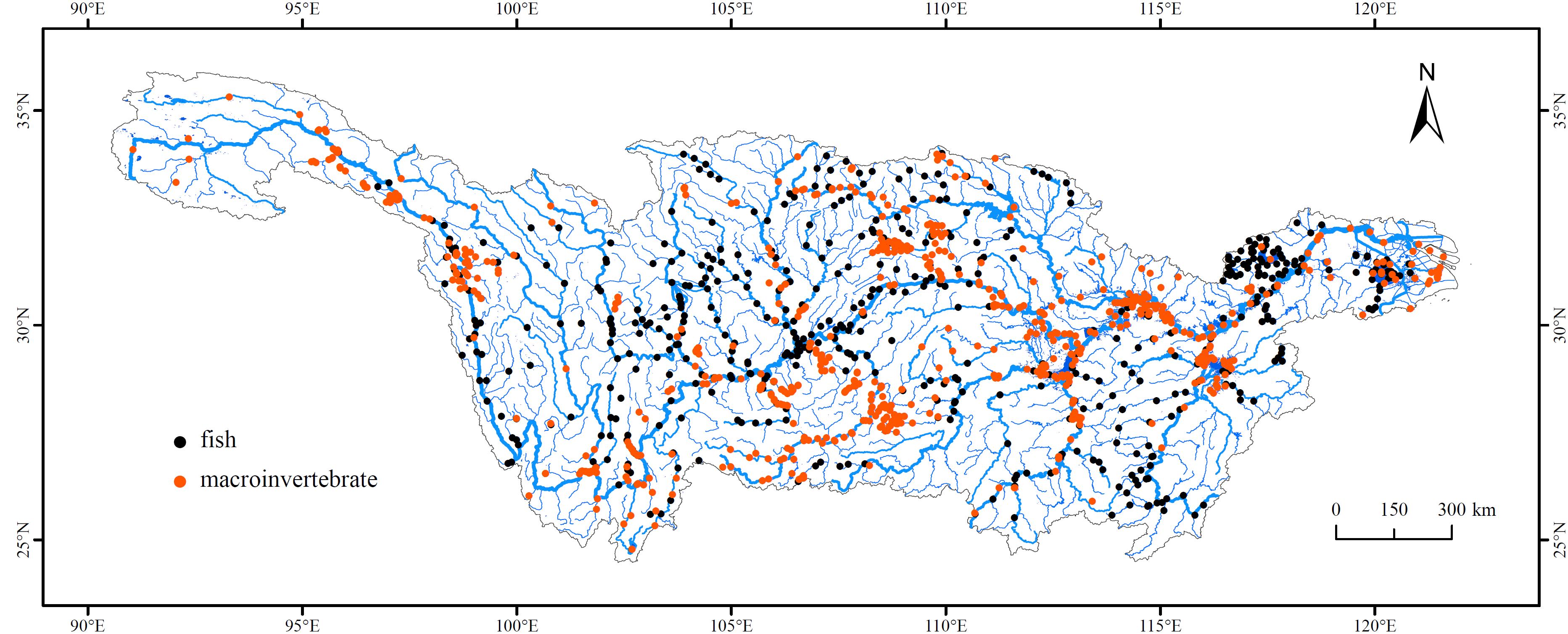


**Appendix 2** Study sections of fish (black dots) and macrozoobenthos (orange dots) in the Yangtze River Basin

### Appendix 3

**Appendix 3** Environmental variables prepared for species distribution models (table)

| **Group** | **Abbreviations** | | | | **Explanation for variables** | **Units** | | |
| --- | --- | --- | --- | --- | --- | --- | --- | --- |
| Bioclimatic variables | bio1 | | | | Annual mean temperature | ℃ | | |
|  | bio2 | | | | Mean monthly temperature range | ℃ | | |
|  | bio3 | | | | Isothermality |  | | |
|  | bio4 | | | | Temperature seasonality |  | | |
|  | bio5 | | | | Max temperature of warmeat month | ℃ | | |
|  | bio6 | | | | Min temperature of coldest month | ℃ | | |
|  | bio7 | | | | Tmperature annual range | ℃ | | |
|  | bio8 | | | | Mean temperature of wettest quarter | ℃ | | |
|  | bio9 | | | | Mean temperature of driest quarter | ℃ | | |
|  | bio10 | | | | Mean temperature of warmest quarter | ℃ | | |
|  | bio11 | | | | Mean temperature of clodest quarter | mm | | |
|  | bio12 | | | | Annual mean precipitation | mm | | |
|  | bio13 | | | | Precipitation of wettest month | mm | | |
|  | bio14 | | | | Precipitation of driest month | mm | | |
|  | bio15 | | | | Precipitation seasonality (CV) |  | | |
|  | bio16 | | | | Precipitation of wettest quarter | mm | | |
|  | bio17 | | | | Precipitation of driest quarter | mm | | |
|  | bio18 | | | | Mean precipitation of warmest quarter | mm | | |
|  | bio19 | | | | Mean precipitation of clodest quarter | mm | | |
|  | wsd | | | | Water surplus and deficit | mm | | |
| Topographic variables | elevation | | | | Elevation | m | | |
|  | slope | | | | Slope | ° | | |
|  | aspect | | | | Aspect | ° | | |
| Hydrological group | area | | | | Catchment area | km^2^ | | |
|  | type | | | | Catchment water body type (1: Mainstem; 2: Primary tributaries; 3: Secondary tributaries; 4: Tertiary tributaries; 5: Quaternary tributaries; 6: Lakes in upper basin; 7: River-isolated lakes; 8: Reservoirs; 9: River-connected lakes) |  | | |
|  | down | | | | Distance from the reach outlet to the final downstream location | km | | |
|  | up | | | | Distance from the reach outlet to the most upstream location | km | | |
|  | flow | | | | Flow accumulation | pixels | | |
| Land cover and population variables | fish | forest | | | Proportion of forest cover | % | | |
|  |  | cultivated | | | Proportion of cultivated cover | % | | |
|  |  | grassland | | | Proportion of grassland cover | % | | |
|  |  | shrubland | | | Proportion of shrubland cover | % | | |
|  |  | wetland | | | Proportion of wetland cover | % | | |
|  |  | water_body | | | Proportion of water_body cover | % | | |
|  | macroinvertebrate | | vegetation | Proportion of vegetation cover | | | % |  |
|  |  |  | water | Proportion of water cover | | | % |  |
|  |  |  | artificial | Proportion of artificial cover | | | % |  |
|  | population | | | | Population per km^2^ | 10^4^/km^2^ | | |
|  | ΔNDVI | | | | Variation of NDVI during 20 yaers | / | | |
| Substrate variables | soilType(1) | | | | Topsoil Texture | / | | |
|  | soilGrav(2) | | | | Topsoil Gravel Content | % | | |
|  | soilSand(3) | | | | Topsoil Sand Fraction | % | | |
|  | soilSilt(4) | | | | Topsoil Silt Fraction | % | | |
|  | soilClay(5) | | | | Topsoil Clay Fraction | % | | |
|  | soilBulk(6) | | | | Topsoil Bulk Density | kg/dm^3^ | | |
|  | soilOrCa(7) | | | | Topsoil Organic Carbon | % | | |
|  | soil pH(8) | | | | Topsoil pH | / | | |
|  | soilCCEC(9) | | | | Topsoil CEC (clay) | cmol/kg | | |
|  | soilCEC(10) | | | | Topsoil CEC (soil) | cmol/kg | | |
|  | soilBaSa(11) | | | | Topsoil Base Saturation | % | | |
|  | soilCaCa(12) | | | | Topsoil Calcium Carbonate | % | | |
|  | soilTEB(13) | | | | Topsoil TEB | cmol/kg | | |
|  | soilGyps(14) | | | | Topsoil Gypsum | % | | |
|  | soilESP(15) | | | | Topsoil Sodicity (ESP) | % | | |
|  | soilECe(16) | | | | Topsoil Salinity (ECe) | dS/m | | |

### Appendix 4


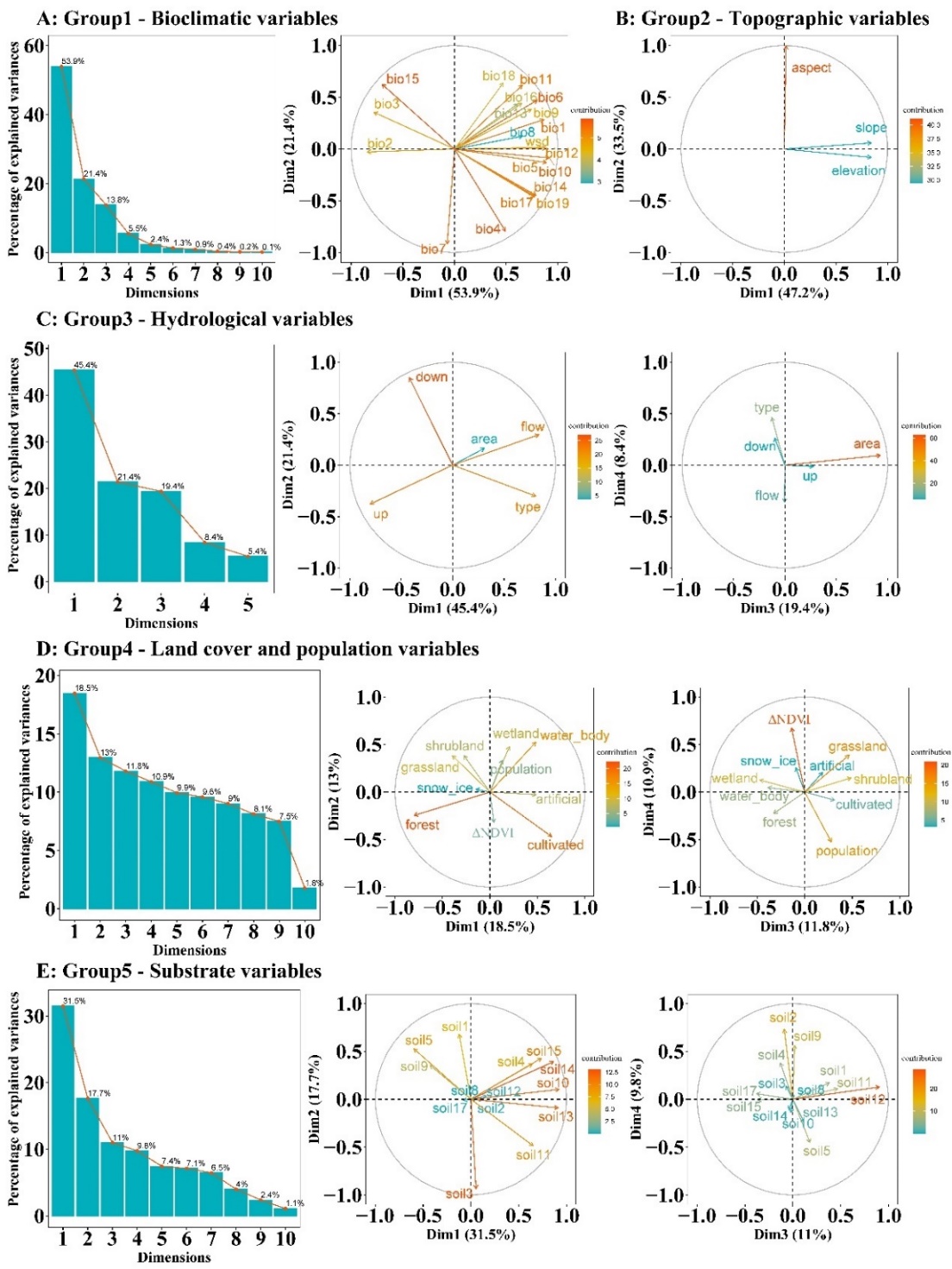


**Appendix 4** Scree plots and biplots in the principal component analysis of five environmental groups for fish. Selected high contribution variables are bio1, bio4, bio5, bio7, bio10, bio12, bio15, wsd, elevation, slope, forest, cultivated, water_body, wetland, shrubland, ΔNDVI, flow, type, down, area, soil2, soil3, soil9, soil10, soil12

### Appendix 5


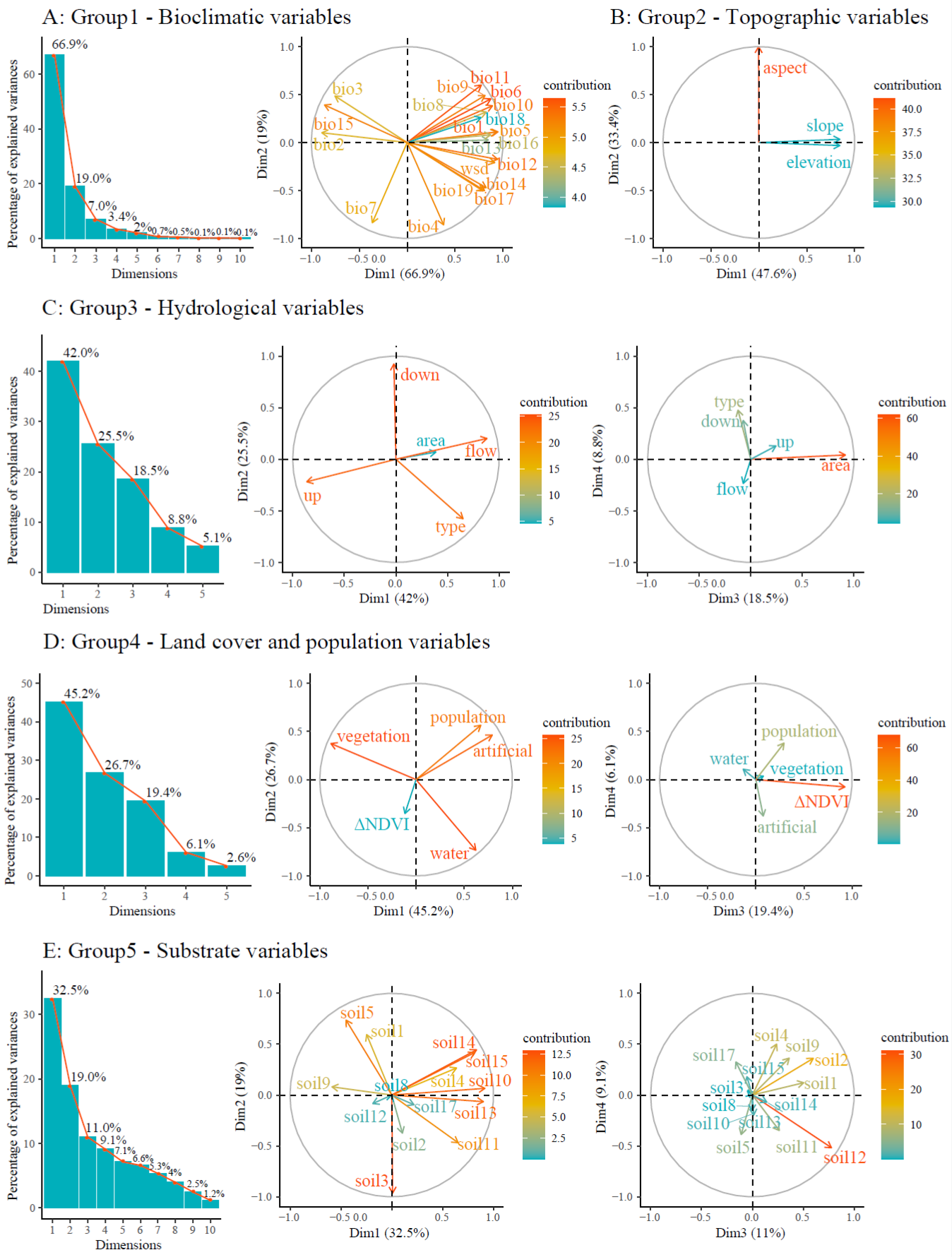


**Appendix 5** Scree plots and biplots in principal component analysis of five environmental groups for macroinvertebrates. Selected high contribution variables are bio4, bio5, bio7, bio10, bio12, wsd, elevation, aspect, vegetation, water, ΔNDVI, population, flow, down, area, type, soil2, soil3, soil5, soil10, soil12.

### Appendix 6

**Appendix 6** Selecting environmental variables for each group in the MaxEnt model.

| Animals | Groups | Environmental variables |
| --- | --- | --- |
| Macroinvertebrates | Naididae | bio10, elevation, aspect, flow, down, type,vegetation, water, ΔNDVI, population, soil3 |
|  | Tubificidae | bio4, bio5, bio7, bio10, bio12, wsd, elevation, flow, down, type, vegetation, water, ΔNDVI, population, soil3, soil5, soil10 |
|  | Hirudinea | bio10, elevation, type, vegetation, water, ΔNDVI, soil10 |
|  | Polychaeta | bio10, elevation, aspect. vegetation, water, ΔNDVI, population, down, type, soil10 |
|  | Prosobranchia | bio10, elevation, down, type, vegetation, water, ΔNDVI, population, soil10, soil12 |
|  | Pulmonata | bio10, elevation, type, vegetation, soil12 |
|  | Bivalvia | bio12, wsd, elevation, down, type, vegetation, water, ΔNDVI, population, soil3, soil10, soil12 |
|  | Malacostraca | bio5, bio10, bio12, wsd, elevation, flow, down, type, vegetation, population, soil2, soil3, soil5 |
|  | Ephemeroptera | bio4, bio5, bio7, bio10, bio12, wsd, elevation, down, area, , type, population, soil3, soil5, soil12 |
|  | Plecoptera | bio5, bio7, bio10, bio12, wsd, elevation, down, area, type, population, soil2, soil3, soil5, soil12 |
|  | Trichoptera | bio4, bio5, bio7, bio10, elevation, area, type, population, soil3 |
|  | other insects | bio4, bio7, elevation, type, population, soil2, soil13 |
|  | Diptera | bio4, bio5, bio10, bio12, wsd, elevation, down, area, type, population, soil5 |
|  | Chironominae | bio4, bio5, bio7, bio10, bio12, wsd, elevation, flow, down, type, vegetation, population, soil3, soil5, soil10 |
|  | Orthocladiinae | bio5, bio7, bio10, bio12, wsd, elevation, down, type, population, soil3, soil5 |
|  | Tanypodinae | bio10, elevation, water, type, vegetation, population, soil10 |
|  | Diamesinae | bio7, bio10, bio12, wsd, elevation, down, type, population, soil2, soil3, soil5 |
|  | others | bio5, bio10, elevation, type, population, soil3, soil5 |
| Fish | DMC | bio1, bio4, bio5, bio7, bio10, bio12, bio15, wsd, elevation, slope, forest, cultivated, water, ΔNDVI, flow, type, down, soil9, soil10, soil12 |
|  | DMH | bio7, bio10, elevation, slope, forest, water, ΔNDVI, flow, type, soil3, soil9, soil10 |
|  | DMO | bio7, bio10, elevation, slope, forest, cultivated, water, flow, type, soil3, soil10 |
|  | DNC | bio1, bio4, bio5, bio7, bio10, bio12, bio15, wsd, elevation, slope, forest, water, flow, type, down, soil12, soil3, soil10 |
|  | DNH | bio4, bio7, bio15, slope, cultivated, bio10, elevation, forest, water, flow, type, soil3, soil10 |
|  | DNO | bio1, bio4, bio5, bio7, bio10, bio12, bio15, elevation, slope, forest, water, flow, type, down, area, soil12, soil3, soil10 |
|  | LMC | bio4, bio7, bio10, elevation, slope, forest, water, cultivated, flow, type, soil3, soil10 |
|  | LMH | bio7, bio10, elevation, slope, forest, water, cultivated, ΔNDVI, flow, type, area, soil3, soil10 |
|  | LMO | bio7, bio10, elevation, slope, forest, water, cultivated, flow, type, soil3, soil10 |
|  | LNC | bio1, bio5, bio7, bio10, elevation, slope, forest, water, ΔNDVI, type, down, flow, soil3, soil10 |
|  | LNH | bio1, bio4, bio5, bio7, bio10, bio12, bio15, wsd, elevation, slope, forest, water, type, cultivated, flow, down, area, soil3, soil10, soil12 |
|  | LNO | bio1, bio4, bio5, bio7, bio10, bio12, bio15, wsd, elevation, slope, forest, water, flow, type, down, soil2, soil3, soil10, soil12 |
|  | UMC | bio7, bio10, elevation, slope, forest, water, ΔNDVI, flow, type, soil3, soil9, soil10 |
|  | UMH | bio7, bio10, elevation, slope, forest, water, wetland, ΔNDVI, flow, type, soil3, soil9, soil10 |
|  | UMO | bio7, bio10, elevation, slope, forest, water, cultivated, wetland, ΔNDVI, flow, type, soil3, soil10 |
|  | UNC | bio1, bio4, bio5, bio7, bio10, bio12, bio15, wsd, elevation, slope, forest, water, flow, type, down, soil12, soil3, soil10 |
|  | UNH | bio7, bio10, elevation, slope, forest, water, ΔNDVI, type, flow, soil9, soil3, soil10 |
|  | UNO | bio1, bio4, bio5, bio7, bio10, bio12, bio15, wsd, elevation, slope, forest, water, wetland, flow, type, down, soil2, soil3, soil9, soil10, soil12 |

NOTE: U, upper; L: lower; D: demersal; M: migratory; N: native; C: carnivore; O: omnivore; H: herbivore

### Appendix 7


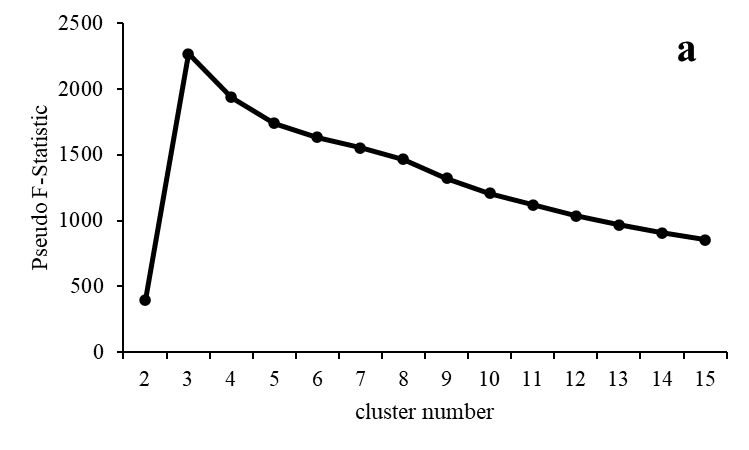

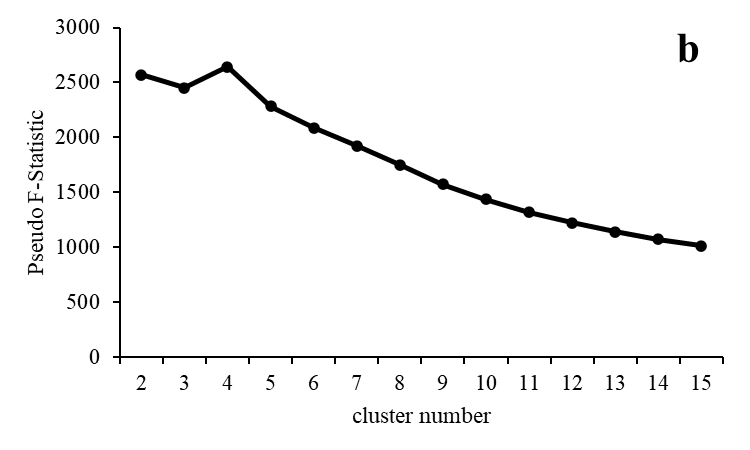

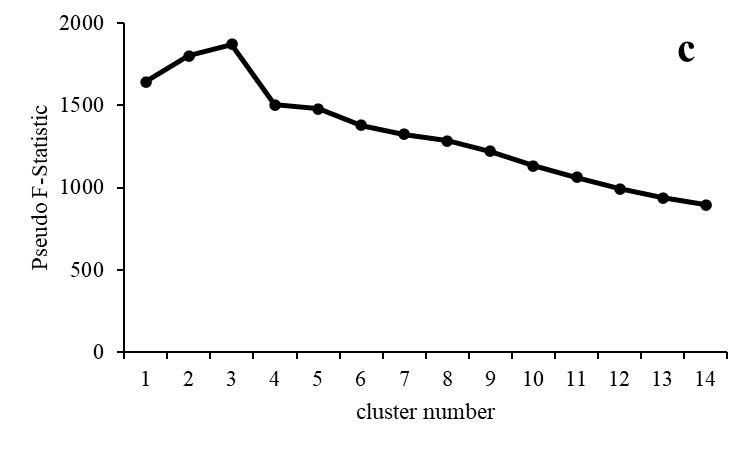


**Appendix 7** The relationship between cluster numbers 2 through 15 and Pseudo F-Statistic. The optimal cluster numbers are three for fish (a), four for macroinvertebrates (b) and four for combined axes of Non-metric multidimensional scaling (NMDS) based on Jaccard dissimilarities (c).

### Appendix 8


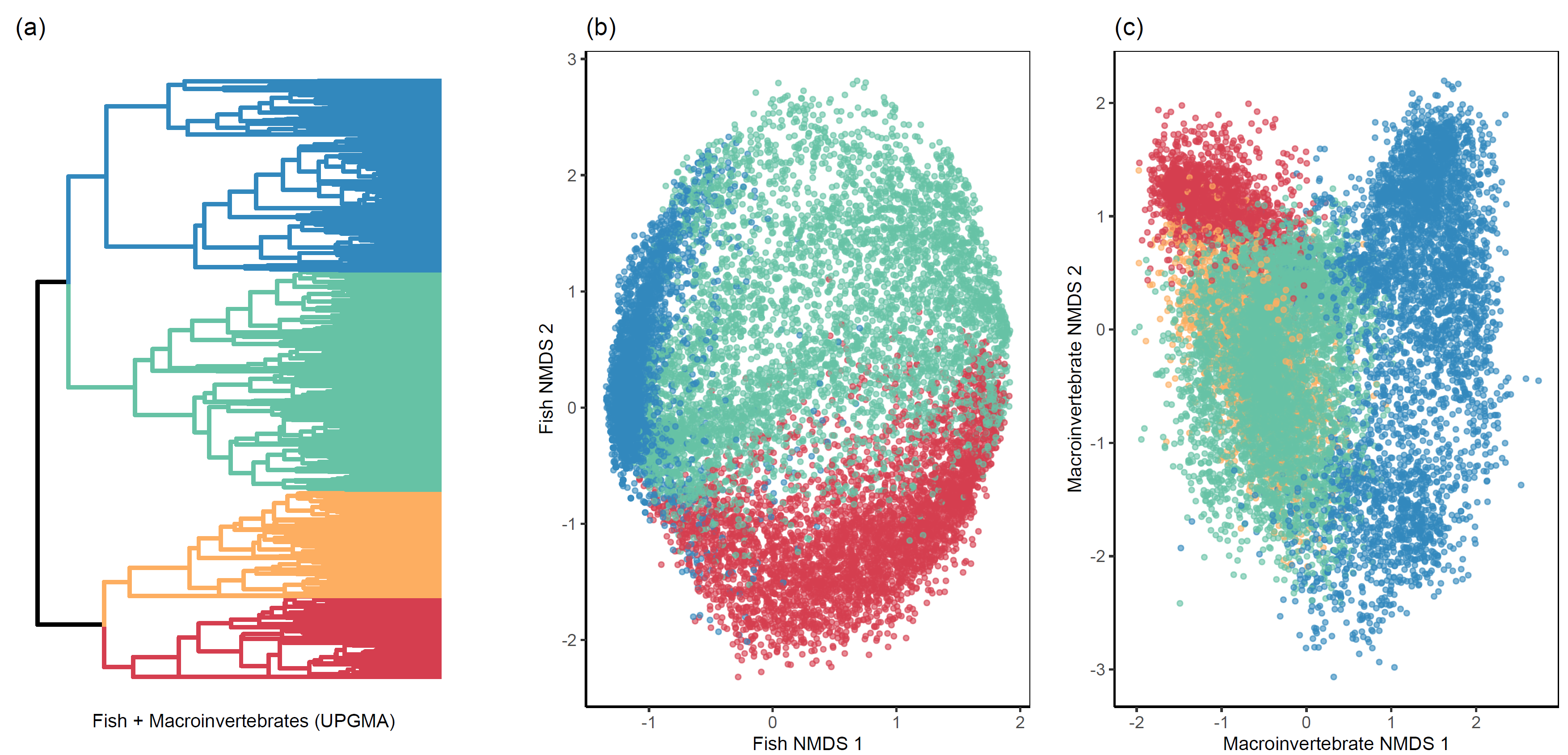


**Appendix 8** UPGMA clustering and NMDS ordination. (a) UPGMA dendrogram based on the first three NMDS axes of combined fish and macroinvertebrate matrix, with color-coded bioregions corresponding to spatial distribution in Figure 3c. (b) NMDS ordination of fish Jaccard dissimilarity showing the three bioregions. (c) NMDS ordination of macroinvertebrate Jaccard dissimilarity showing the four bioregions. The three and four clusters in NMDS ordinations are corresponded to the optimal cluster numbers in spatial clustering.

### Appendix 9

**Appendix 9** Results of PERMANOVA and pairwise PERMANOVA tests based on Bray–Curtis dissimilarities among the four groups.

| **Comparison** | **Df** | **Sum of Squares** | ***R²*** | **F.Model** | **P (adj)** | **Significance** |
| --- | --- | --- | --- | --- | --- | --- |
| **Overall PERMANOVA** | 1 | 17374.49 | 0.211 | 3673.20 | 0.001 | ****** |
|  | 13714 | 64868.10 | 0.789 |  |  |  |
|  | **Total** | 82242.60 | 1.000 |  |  |  |
| **Pairwise comparisons** |  |  |  |  |  |  |
| HWS vs HDS |  |  | 0.121 | 839.75 | 0.001 | ****** |
| HWS vs DQW |  |  | 0.174 | 1564.52 | 0.001 | ****** |
| HWS vs MLF |  |  | 0.335 | 3893.62 | 0.001 | ****** |
| HDS vs DQW |  |  | 0.074 | 481.31 | 0.001 | ****** |
| HDS vs MLF |  |  | 0.292 | 2605.68 | 0.001 | ****** |
| DQW vs MLF |  |  | 0.168 | 1539.44 | 0.001 | ****** |

Note: Headwater and Western Sichuan Plateau, HWS; Hengduan Mountains-Sichuan Basin, HDS; Dabie-Qinling-Wuling Mountains, DQW; and Mid-lower Floodplain region, MLF

### Appendix 10


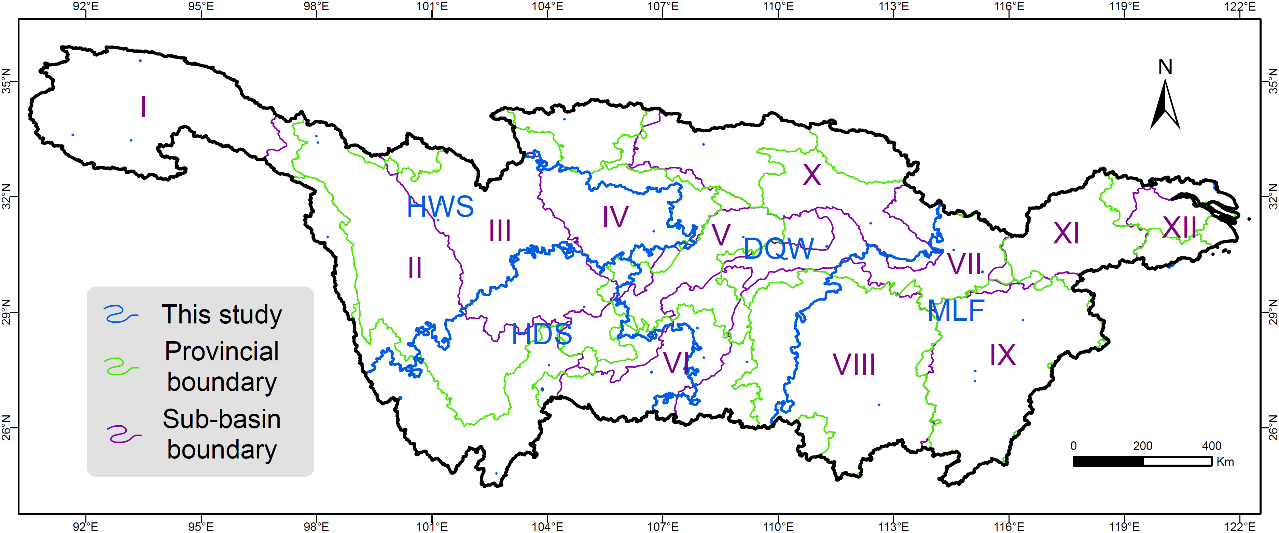


**Appendix 10** Comparison among provincial boundaries, sub-basin boundaries, and the bioregional delineation derived from this study.

Headwater and Western Sichuan Plateau, HWS; Hengduan Mountains-Sichuan Basin, HDS; Dabie-Qinling-Wuling Mountains, DQW; and Mid-lower Floodplain region, MLF

I: The headwater sub-basin; II: Jinshajiang sub-basin; III: Mintuo sub-basin; IV: Jialingjiang sub-basin; Ⅴ: Upper mainstem sub-basin; Ⅵ: Wujiang sub-basin; Ⅶ: Middle mainstem sub-basin; VIII: Dongtinghu sub-basin; IX: Poyanghu sub-basin; X: Hanjiang sub-basin; XI: Lower mainstem sub-basin; XII: Taihu sub-basin).
