# Supplementary material for: Delineating Diversity‐Based Freshwater Bioregions by Integrating Fish and Macroinvertebrates With Species Distribution Models and Spatial Clustering
Source: Ecol Evol. 2025 Dec 8;15(12):e72609. doi: 10.1002/ece3.72609 (PMC12683362; doi:10.1002/ece3.72609)
Supplement: Supplementary file 1 — Appendix S1: Hydrological network of rivers and lakes in the Yangtze River Basin. Appendix S2: Study sections of fish (black dots) and macrozoobenthos (orange dots) in the Yangtze River Basin. Appendix S3: Environmental variables prepared for species distribution models (table). Appendix S4: Scree plots and biplots in the principal component analysis of five environmental groups for fish. Appendix S5: Scree plots and biplots in principal component analysis of five environmental groups for macroinvertebrates. Appendix S6: Selecting environmental variables for each group in the MaxEnt model. Appendix S7: The relationship between cluster numbers 2 through 15 and pseudo F‐statistic. Appendix S8: UPGMA clustering and NMDS ordination. Appendix S9: Overall PERMANOVA and pairwise PERMANOVA tests based on Bray–Curtis dissimilarities among the four groups. Appendix S10: Comparison of provincial boundaries, sub‐basin boundaries, and the bioregional delineation in this study. Appendix S11: The ODMAP protocol of this study. [file ECE3-15-e72609-s001.zip › Appendix 11.docx]

Bioregionalization for biodiversity conservation in freshwater ecosystem: a quantitative and representative method

– ODMAP Protocol –

Yajing He, Hongzhu Wang, Junyan Wu, Yongjing Zhao, Wenjuan Gao, Yongde Cui

2024-07-22

## Overview

#### Authorship

Contact : [heyajing@ihb.ac.cn](mailto:heyajing@ihb.ac.cn)

<Study link>

#### Model objective

Model objective: Forecast and transfer

Target output: continuous habitat suitability index, abundance

#### Focal Taxon

Focal Taxon: fish and macroinvertebrate

#### Location

Location: the Yangtze River Basin

#### Scale of Analysis

Spatial extent: 90.545568678114, 122.245568676846, 24.475, 35.899999999543 (xmin, xmax, ymin, ymax)

Spatial resolution: 0.03 km

Temporal extent: 1980-2017

Boundary: river-lake network

#### Biodiversity data

Observation type: field survey, standardised monitoring data

Response data type: presence-only, richness

#### Predictors

Predictor types: climatic, habitat, topographic, edaphic, hydrological, social

#### Hypotheses

Hypotheses: Each species occupies a specific ecological niche - a unique combination of resources and environmental conditions that allow it to survive and reproduce. Species-environment relationships are shaped by how well a species’ adaptations match its niche.

#### Assumptions

Model assumptions: Species Have Identifiable Niches: Niche theory and related hypotheses assume that each species has a definable and measurable ecological niche.

#### Algorithms

Modelling techniques: maxent

<Model complexity>

<Model averaging>

#### Workflow

Model workflow: The environmental variables of each section were extracted based on the coordinates of latitude and longitude using the tool “extract by mask” in ArcGIS 10.6. Principal components analysis (PCA) was applied to reduce the dimension within environmental groups. To avoid overfitting of the model results to one group, principal components with a cumulative variance explanation ratio exceeding 70% were selected according to the scree plots, and variables with high loadings of each principal component were extracted. Then, the Mantel test was used to analyze the relationship between environmental variable matrix and the species composition matrix of fish and macroinvertebrate, and to determine the environmental variables for different groups to construct modeling.

Distribution of fish species and macroinvertebrate taxa and corresponding high loading variables were entered into MaxEnt 3.3.3 k, which was the most commonly used and reliable SDMs. The model randomly selected 75% data for training and 25% for testing. In the MaxEnt, the area under the curve (AUC) of the Receiver Operating Characteristic (ROC) curve was used to evaluate model accuracy. Models were acceptable when the values of AUCs were above 0.8. We reclassified the potential probability of acceptable models into binary data (0~0.5 to 0 and 0.5~1 to 1). Then, we calculated the total grid values of each HU, and the higher value indicated more suitable HUs for species. Sort the total value based on quantile (Q), and divide the HUs into five categories: extremely low suitable (0), low suitable (0, Q1], medium suitable (Q1, Q2], high suitable (Q3, Q4], and extremely high suitable (> Q4). Then high and very high suitable HUs were regarded as the potential presence distributions to fish or macroinvertebrates. In addition, for some species with insufficient distribution sites and those failed in constructing models, we took their real distribution sites as presence.

#### Software

Software: MaxEnt 3.3.3

<Code availability>

<Data availability>

## Data

#### Biodiversity data

Taxon names: A total of 391 fish species in 141 genera, 35 families and 15 orders are included in this analysis. Among those, the top five of order richness are Cypriniformes (76%, 298 species), Siluriformes (10%, 38 species), Gobiiformes (3%, 13 species), Centrarchiformes (2%, 8 species) and Osmeriformes (1%, 6 species). A total of 984 taxa of macroinvertebrate belonging to 608 genera, 181 families, 32 orders, 9 classes and 5 phyla are included in this study. Among those, Arthropoda is the most abundant phylum (52%, including 515 taxa and 477 genera), followed by Mollusca (35%, including 339 species and 71 genera) and Annelida (35%, including 128 species and 58 genera).

<Taxonomic reference system>

Ecological level: communities, taxonomic groups, functional group

Data sources: For macroinvertebrates, a total of 637 sections (each lake and each river segment is considered a single section) are included in this study, of which 284 sections of macroinvertebrate data are collected in the fields by our research group (from 1980 to 2017) by D-net, Surber net in the rivers and Petersen grab in the lakes. A total of 298 articles from 1984 to 2017 were considered. Filtering researches from authoritative institutes and with similar study methods, 143 articles were screened to acquire data.

The original data of fish distributions partly (235 sections) came from field collection, and partly (346 sections) were from literatures and books (1984 to 2017) .

Sampling design: random

Sample size: For macroinvertebrates, a total of 637 sections (each lake and each river segment is considered a single section) are included in this study. The original data of fish distributions partly (235 sections) came from field collection, and partly (346 sections) were from literatures and books (1984 to 2017) .

Clipping: China/the Yangtze River Basin

Cleaning: the distribution of taxa less than 10 sections will be excluded.

<Absence data>

Background data: In the original data, oligochaetes and mollusks were identified into species or genera, while insects and other groups were identified into genera or families. We classified the data from the previous step into genus (95%) or families (5%) level for species distribution models. We compiled fish catalogs, functional traits, etc., based on databases such as the World Fish Database (<https://www.fishbase.de/>), or authoritative books. Fish ecological guilds included feeding habits (carnivore, omnivore, herbivore), migratory habits (migratory, native) and depth range (upper, lower, demersal). According to the three habits above, fish was classified into eighteen groups. The conservation status (national or provincial), endangerment category (Critically Endangered, CR; Endangered, EN; Vulnerable, VU; Near Threatened, NT; Least Concern, LC; Data Deficient, DD) and endemism (endemic to China or YRB) are referred to the book China’s Red List of Biodiversity: Vertebrates, Volume V, Freshwater Fishes (2021 edition) .

#### Data partitioning

Training data: 75%

<Validation data>

Test data: 25%

#### Predictor variables

Predictor variables: 1) Bioclimatic variables, mainly including parameters and variables relaxant to temperature and precipitation. Bio1~bio19 downloaded from the World Climate Database (<http://www.worldclim.org>) . Water surplus and deficit were calculated by subtracting the annual mean precipitation from the annual mean water evaporation.Water evaporation data were obtained from the DIVA-GIS website (<http://www.diva-gis.org/>). 2) Topographic variables, mainly including elevation, slope and aspect, were obtained from the website of the Food and Agriculture Organization of the United Nations (<http://www.fao.org>). 3) Hydrological variables, included HUs area, water body type, downstream distance, upstream distance and flow accumulation. Except water body type, others were from databases of HydroBASINS, HydroRIVERS and HydroLAKES. 4) Social variables, including the proportion of vegetation, water and artificial land, variation of NDVI during twenty years (1998~2018), and the population density per square kilometer. All data in this group were from the Resource and Environment Data Cloud Platform (<http://www.resdc.cn>). We calculated the proportion of the total vegetation, wetlands and waters, and artificial land area in each hydrological unit. 5) Substrate variables, mainly including topsoil texture and other characteristics, were obtained from the Harmonized World Soil Database (FAO/IIASA/ISRIC/ISSCAS/JRC, 2012) . The raster boundary of environmental variables extracted by the mask of the YBR layer with the raster resolution of 30 arc-seconds, and the coordinate system was set to WGS-1984.

Data sources: 1. <http://www.worldclim.org> 2. <http://www.diva-gis.org/> 3. <http://www.fao.org> 4. HydroBASINS, HydroRIVERS and HydroLAKES 5. <http://www.resdc.cn> 6. FAO/IIASA/ISRIC/ISSCAS/JRC, 2012

Spatial extent: 90.545568678114, 122.245568676846, 24.475, 35.899999999543 (xmin, xmax, ymin, ymax)

Spatial resolution: 0.03 km

Coordinate reference system: GCS_WGS_1984

<Temporal extent>

Temporal resolution: 2000-2020

Data processing: by the tool of “Georeferencing” and “Projection Transformation”

Dimension reduction: We divided environmental variables into groups, and use PCA and Mantel test to reduce them.

#### Transfer data

<Data sources>

<Spatial extent>

<Spatial resolution>

<Temporal extent>

<Models and scenarios>

<Quantification of Novelty>

## Model

#### Variable pre-selection

Variable pre-selection: The environmental variables of each section were extracted based on the coordinates of latitude and longitude using the tool “extract by mask” in ArcGIS 10.6. Principal components analysis (PCA) was applied to reduce the dimension within environmental groups. To avoid overfitting of the model results to one group, principal components with a cumulative variance explanation ratio exceeding 70% were selected according to the scree plots, and variables with high loadings of each principal component were extracted. Then, the Mantel test was used to analyze the relationship between environmental variable matrix and the species composition matrix of fish and macroinvertebrate, and to determine the environmental variables for different groups to construct modeling.

#### Multicollinearity

Multicollinearity: PCA and Mantel test

#### Model settings

<maxent>

<Model settings (extrapolation)>

#### Model estimates

Coefficients: Models were acceptable when the values of AUCs were above 0.8

Parameter uncertainty: only substrate variables are resampled by Kriging interpolation method

Variable importance: Do jackknife to measure variable importance

#### Model selection - model averaging - ensembles

Model selection: according to the data type

#### Analysis and Correction of non-independence

<Spatial autocorrelation>

#### Threshold selection

<Threshold selection>

## Assessment

#### Performance statistics

Performance on training data: AUC

Performance on validation data: AUC

Performance on test data: AUC

#### Plausibility check

Response shapes: Marginal Response Curves and single Variable Response Curves

Expert judgement: check the distribution of suitability

## Prediction

#### Prediction output

Prediction unit: river-lake network

Post-processing: clipping

#### Uncertainty quantification

<Scenario uncertainty>

<Novel environments>
